# Extrusion Processing of Rapeseed Press Cake-Starch Blends: Effect of Starch Type and Treatment Temperature on Protein, Fiber and Starch Solubility

**DOI:** 10.3390/foods10061160

**Published:** 2021-05-21

**Authors:** Anna Martin, Susanne Naumann, Raffael Osen, Heike Petra Karbstein, M. Azad Emin

**Affiliations:** 1 Department of Food Process Development, Fraunhofer Institute for Process Engineering and Packaging IVV, 85354 Freising, Germany; susanne.naumann@ivv.fraunhofer.de; 2 Singapore Institute of Food and Biotechnology Innovation, Agency for Science, Technology and Research (A*STAR), Singapore 138669, Singapore; Raffael_Osen@sifbi.a-star.edu.sg; 3 Institute of Process Engineering in Life Sciences, Chair of Food Process Engineering, Karlsruhe Institute of Technology, 76131 Karlsruhe, Germany; heike.karbstein@kit.edu (H.P.K.); azad.emin@kit.edu (M.A.E.)

**Keywords:** canola, protein solubility, dietary fiber, starch gelatinization, extrusion, expansion, biopolymers, closed-cavity rheometer

## Abstract

For the valorization of oilseed press cakes into food products, extrusion can be used. A common way of applying the protein- and fiber-rich press cakes in directly expanded products is the combination thereof with starch, since starch gives a favourable texture, which correlates directly to expansion. To control product properties like expansion of protein and fiber-rich extruded products, the underlying physicochemical changes of proteins, fibers and starch due to thermomechanical input need to be comprehensively described. In this study, rapeseed press cake (RPC) was extruded and treated under defined thermomechanical conditions in a closed-cavity rheometer, pure and in combination with four starches. The impact of starch type (potato PS, waxy potato WPS, maize MS, high-amylose maize HAMS) and temperature (20/25, 80, 100, 120, 140 °C) on protein solubility, starch gelatinization (D_gel_), starch hydrolysis (S_H_) and fiber solubility of the blends was evaluated. The extrusion process conditions were significantly affected by the starch type. In the extruded blends, the starch type had a significant impact on the protein solubility which decreased with increasing barrel temperature. Increasing barrel temperatures significantly increased the amount of soluble fiber fractions in the blends. At defined thermomechanical conditions, the starch type showed no significant impact on the protein solubility of the blends. Therefore, the observed effects of starch type on the protein solubility of extruded blends could be attributed to the indistinct process conditions due to differences in the rheological properties of the starches rather than to molecular interactions of the starches with the rapeseed proteins in the blends.

## 1. Introduction

The valorization of food production by-products into directly expanded food products using extrusion has been a well-established and well-studied technology in the past years [1,2,3,4,5,6,7,8,9]. In response to the consumer demand for healthy and sustainable products, protein- and fiber-rich by-products of the food production chain have been utilized in order to aim at beneficial nutritional profiles, as well as designated texture properties [10]. A promising source of protein and fiber is rapeseed press cake (RPC), which constitutes the residue after the oil-pressing of rapeseeds. Due to its high availability and some nutritional limitations that restrict its inclusion level, RPC is relatively inexpensive compared to other press cakes like soybean or sunflower. RPC has been applied as an ingredient in blends in order to investigate its impact on the extruder response, rheological properties, and physical quality properties of extruded food and feed [11,12,13,14,15]. However, its maximum level of incorporation, especially in extruded food products, is restricted due to two main factors—the presence of antinutritional components (ANFs) and its effect on product quality, such as texture properties. To overcome the hurdle of large quantities of ANFs in RPC, a number of studies have investigated the impact of different treatments on ANF reduction and have successfully accomplished a significant reduction of glucosinolates, tannins, phytic acids and raw fiber [16,17,18,19,20]. It can therefore be assumed that in the next few years, the use of RPC with reduced ANF levels will allow the production of RPC-based extruded products for human consumption. The second major restriction of using RPC in extruded products is the limited knowledge of its effect on product quality, particularly on the product texture that is defined by its expansion properties. Expansion is known to be driven by the sudden exceedance of water vapor pressure at the extruder die exit. Expansion properties highly depend on the extrusion process parameters, like barrel temperature, moisture content or screw speed, that in turn influence the rheological, chemical and physicochemical properties of the extruded matrix.

High expansion properties of extruded snacks are mostly generated by extrusion processing of starch-based formulations, such as potato, rice, wheat or maize starch [21]. When RPC (10–70 g/100 g) was added to starch blends in previous studies, severe changes in expansion properties, as well as in rheological and physicochemical properties were monitored. In our recent study [15] we reported that up to 70 g/100 g RPC could be implemented in potato starch blends, resulting in a high degree of expansion. However, a combination of maize starch and RPC (10–40 g/100 g) resulted in a lower degree of expansion, and the application of RPC resulted in changes of rheological and physicochemical properties of the blends and the corresponding extruded samples, like viscous and elastic properties, water absorbance or water solubility [14]. In these studies, the underlying physicochemical transformations of RPC and starch have not been described yet, but can be related to a number of heat- and shear-induced reactions that take place due to thermomechanical treatment.

RPC exhibits protein content between 19–40 g/100 g [22,23,24]. In general, plant protein fortification of starch-based extruded products has been reported to result in significant changes of the final product properties due to unfolding, realigning, hydrolysation, denaturation and cross-linking of the proteins with each other or with other ingredients, like starch, sugar or dextrin molecules [25,26]. Denaturation and aggregation of plant proteins induced by thermomechanical input has been reported to reduce the protein solubility in water or salt buffers [27]. In particular, Zhang et al. [28] reported that extrusion-processing of rapeseed protein meal resulted in significant protein aggregation, and consequently in a lower amount of extractable protein compared to untreated samples. Matthey and Hanna [29] proposed that protein–starch interactions can in turn inhibit the degradation of starch during extrusion, especially because proteins adjust the water distribution in the melt. Starch degradation, indicated by the degree of gelatinization, is known to be affected by the botanical origin, as well as by the amylose/amylopectin ratio of the starch type [30]. Some studies suggest that amylopectin and amylose may physically interact or form new bonds with, for example, proteins during thermomechanical treatment, wherefore product properties like expansion are influenced [31]. Therefore, it can be expected that with the combination of RPC and starch in blends, reactions of rapeseed protein and the competition for water during thermomechanical treatment between starch and RPC will affect the degree of starch degradation and the solubility of rapeseed proteins.

Besides proteins, RPC exhibits about 36 g/100 g of total dietary fiber content, whereof 88 g/100 g are soluble and 12 g/100 g are insoluble [15]. A transformation of rapeseed fiber components from insoluble to soluble can be expected, as reported in previous studies [32,33,34]. This would in turn have an effect on the rheological properties, and consequently on the final product properties of extruded RPC/starch products.

In order to increase the amount of RPC in starch-based extruded snacks and to control the quality of the extruded products, the underlying physicochemical changes of these biopolymers need to be investigated in relation to the applied process conditions.

Therefore, in this study, the impact of extrusion processing on the physicochemical properties of rapeseed protein, rapeseed fiber, as well as of starch was evaluated. RPC as a pure component and combined in blends with four starch types was investigated, both untreated and after thermomechanical treatment.

It was expected that the extruder response (SME and product temperature) during extrusion would be affected by the blend composition of the starch type and the resulting rheological properties, respectively, although the process conditions (barrel temperature, mass flow rate, screw speed) were kept constant. Therefore, to overcome the hurdle of indistinct temperatures and shear rates applied to the blends in the extruder, a closed-cavity rheometer was used in order to execute thermomechanical treatments at defined temperatures and shear rates.

## 2. Materials and Methods

### 2.1. Material and Preparation of Blends

Cold-pressed 00-type RPC was kindly provided by Teutoburger Ölmühle (Ibbenbüren, Germany). The temperature during pressing did not exceed 60 °C. The pH of RPC was 5.97 ± 0.25. Potato starch (PS) and waxy potato starch (WPS Eliane^TM^ 100) were kindly provided by Avebe (Veendam, Netherlands). Maize starch (MS) and high-amylose maize starch (HAMS) (Hylon^TM^ VII PCR) were kindly provided by Ingredion (Hamburg, Germany). The amylose content of the starches, as reported in the specifications of the suppliers, were 25, 1, 26 and 67 g/100 g for PS, WPS, MS and HAMS. The RPC was milled to <500 µm before it was mixed in a 70:30 ratio wet basis (w.b.) with PS, WPS, MS or HAMS in a Spiral-Mixer SP 12 (DIOSNA Dierks & Söhne GmbH, Osnabrück, Germany) for 60 min, followed by an incubation at 20 °C for at least 8 h. Prior to extrusion, the dry matter content of the mixtures was analyzed (MA 40, Sartorius AG, Göttingen, Germany) as described in the German Food Act [35].

The moisture content of the materials and extrudates was determined according to the German Food Act [35]. The protein content was analyzed based on the Dumas method according to the German Food Act [35] using a TruMac N Protein Analyzer (LECO, St. Joseph, MI, USA). The ash content was determined according to the AOAC International method 945.46 [36]. The crude fiber content was determined according to the AOAC International method 962.09 [37]. The starch content was determined as previously described [38]. Water absorption (g/g) of the raw materials and extruded samples was analyzed according to the AACC method 56–20.01, and water solubility (%) was determined as previously described [39]. The particle sizes of MS, RPC and RP were determined using a Malvern Mastersizer S Long Bed Version 2.15 laser diffraction particle size analyzer (Malvern Instruments, Malvern, UK) as previously described [40]. Analyses were carried out in duplicate.

### 2.2. Extrusion Processing

The extrusion process was carried out using a 26 mm pilot scale twin screw co-rotating extruder with a L/D ratio of 25/1 (ZSK 26 Mc, Coperion, Stuttgart, Germany). The mass flow rate was kept at 10 kg/h, the moisture content of the melt was set to a 29 g/100 g dry matter basis (d.m.), the screws rotated with 300 rpm, and the temperature of the last barrel segment was set to 20, 80, 100, 120 or 140 °C. A detailed temperature and screw profile can be found in Table 1. After extrusion, the samples were dried in an oven (Thermo Scientific Heraeus UT 6760, Thermo Electron LED GmbH, Langenselbold, Germany) at 40 °C for 24 h, milled at 14.000 rpm to <500 µm (ZM 200, Retsch, Haan, Germany), vacuum-sealed and stored at 20 °C until further analyses.

### 2.3. Thermomechanical Treatment at Defined Temperatures and Shear Rates

Defined thermomechanical conditions were applied using a closed-cavity rheometer (RPA elite, TA instruments, New Castle, DE, USA). The cavity can be pressurized (4.5 MPa) and sealed and the device allows the analyses of low moisture samples at high temperatures without water vaporization or material slippage [41]. Before analyses, the samples were brought to 29 g/100 g d.m. moisture content by mixing them with deionized water in a Thermomix (Vorwerk, Wuppertal, Germany). Afterwards, the samples were incubated in a fridge (4 °C) for at least 24 h to ensure homogeneous water distribution. For rheological analyses, 6 g of each sample were brought to room temperature and placed on the cone.

Extrusion-like conditions were simulated by applying temperatures corresponding to the barrel temperatures during extrusion. Isothermal time sweep tests at 25, 80, 100, 120 or 140 °C were performed at a shear rate of 50 s^−1^ (corresponding to f = 10 Hz and γ = 80%, non-LVE region). The treatment time was 60 s to mimic the residence time in the used extruder. Since the temperature in the measuring chamber of the closed-cavity rheometer is cooled by air, the lowest possible treatment temperature was 25 °C. For each treatment, at least five samples were collected. After treatment, the samples were treated as the extruded samples (see Section 2.2) and investigated in regard of the extractable protein content and protein solubility (see Section 2.4.1).

### 2.4. Physicochemical Properties

#### 2.4.1. Extractable Protein Content and Protein Solubility

The content of the salt-soluble protein fraction in the untreated and thermomechanically treated samples in NaCl was determined at pH 4, 7 and 11 and is further referred to as extractable protein content. Generally, salt buffers have been used in previous research in order to extract protein in its native state [43].

1500, 2000 or 3000 mg of each sample were placed in 50 mL beakers. NaCl of 0.1 M was added, and the pH was adjusted as desired with NaOH for alkaline samples and HCl for acidic samples. The samples were placed in a magnetic stirring plate at 200 rpm for 1 h. After 30 min and after 1 h, the pH was checked and adjusted if necessary. Each sample was placed in a 50 mL volumetric flask and filled up to the mark with 0.1 M NaCl.

After the protein extraction step, 20 mL of the solution were placed in centrifuge tubes and centrifuged for 15 min at 15 °C and 20.000 rpm. The supernatant was filtered (Whatman^TM^, diameter 150 mm, pore size 4–12 μm) and stored at −20 °C until analysis.

The Dumas method established by Eblinger et al. [44] was employed to determine the protein content in the supernatants based on its dry matter. Analyses were carried out in triplicate.

The protein solubility was calculated using Equation (1). V represents the initial volume of the sample (50 mL), PC refers to the protein content present in the supernatant as determined by the Dumas method, m_s_ refers to the initial mass of the sample, DM_s_ is the dry matter of the sample, and PC_dm_ is the protein content of the dry matter of the sample.

Psol = (V × PCsupernatant × 100)/(ms × DMs × PCdm)(1)

#### 2.4.2. Starch Gelatinization

The gelatinization temperature T_gel_ and enthalpy ∆ H of RPC/starch blends were analyzed by using a differential scanning calorimeter (DSC) (Q2000, TA Instruments, New Castle, DE, USA). Slurries were prepared with the unextruded and extruded samples to obtain a final moisture content of 60 g/100 g (w.b.) using a Thermomix (Vorwerk, Wuppertal, Germany). Slurry amounting to 15–20 g were placed into aluminum pans and sealed for analysis. A sealed empty pan was used as a reference. A heating ramp of 20–120 °C with a heating rate of 10 °C/min was applied for one scan. Analysis was carried out using the TA Universal Analysis software (4.4 A, TA Instruments, New Castle, DE, USA). T_gel_ was taken as the peak temperature of the gelatinization endotherm. The degree of gelatinization D_gel_ was calculated according to Equation (2), where ∆ H_0_ refers to the gelatinization enthalpy of the unextruded formulation and ∆H_gel_ indicates the gelatinization enthalpy of the extruded formulations. Analysis was carried out in triplicate.

D_gel_ = (∆H_0_ − ∆H_gel_)/∆H_0_ × 100 (2)

#### 2.4.3. Starch Hydrolysis

The total hydrolyzed starch of RPC/starch blends was analyzed by using a Starch UV Test kit (r-biopharm, Darmstadt, Germany) according to Beutler et al. [38]. Starch was hydrolyzed using amyloglucosidase, hexokinase and glucose-6-phosphate dehydrogenase. Analysis was carried out at least in duplicate for unextruded samples and samples extruded at 100 °C or 140 °C.

#### 2.4.4. Soluble and Insoluble Dietary Fiber Analysis

Soluble and insoluble fiber contents of non-extruded RPC and RPC/starch blends were analyzed by enzymatic-gravimetric analysis according to AOAC 991.43. Analyses were carried out in triplicate.

#### 2.4.5. Statistical Analysis

Data were analyzed using OriginPro (2018 b, OriginLab Corporation, Northampton, MA, USA) by means of two-way analysis of variance (ANOVA). Statistics were considered significant when *p* ≤ 0.05. When appropriate, means were compared using Tukey’s honest significance test.

## 3. Results and Discussion

### 3.1. Chemical Analysis

Table 2 reports the chemical composition of RPC and RPC/starch blends. The RPC/starch blends exhibited very similar protein, ash, starch and lipid contents. TDF content increased in the order WPS30/RPC70, PS30/RPC70, MS30/RPC70 and HAMS30/RPC70. MS blends exhibited the highest, and PS blends the lowest amount of SDF. HAMS blends had a significantly higher amount of IDF compared to blends with PS, MS und WPS that showed the least amount of IDF.

The effect of starch type on the amounts of dietary fiber in untreated samples is likely to be an effect of the analysis method and the presence of resistant starch. Dietary fiber analysis in this study was initiated with an enzymatic starch digestion step (initiated by amylase) at the beginning of the extraction, and resistant starches, if present, remain unaffected by this treatment. It is known from previous studies that uncooked high-amylose starches are more resistant to enzymatic hydrolysis than high-amylopectin starches [45].

### 3.2. Extruder Response

Table 3 reports the extruder response as a function of barrel temperature and starch type generated by the RPC/starch blends. The addition of starch to RPC had a significant effect on the SME and product temperature. At a T_B_ of 20 °C, the product temperature increased with starch addition irrespective of starch type. At a T_B_ of 80 °C and 120 °C, RPC generated a similar product temperature as HAMS30/RPC70 and WPS30/RPC70. RPC as a single component could not be extruded at a T_B_ of 140 °C due to severe extruder clogging. We assume that this was due to the high water absorption of fiber components in RPC.

A comparison of all RPC/starch blends exhibited that starch type and T_B_ had a significant impact on SME (*p* < 0.01). At all T_B_, RPC/WPS blends exhibited the highest SME values, and RPC/MS blends the lowest ones.

RPC/PS and RPC/WPS blends exhibited higher SME at all T_B_ than RPC/MS and RPC/HAMS blends. With increasing T_B_, the SME significantly decreased in all RPC/starch blends, which was due to a temperature-induced decrease of melt viscosity. PS and WPS gelatinized at a lower temperature compared to maize starches (see Section 3.5). This may have resulted in the release of amylose and amylopectin molecules in PS/WPS at a lower T_B_ compared to MS and HAMS increasing the SME.

Furthermore, the crystalline regions of the starches can act as “rigid structures” during extrusion, resulting in a more pronounced friction in the melt or between the melt and the extruder barrel [46]. Since WPS blends exhibit the highest amylopectin content and therefore a higher number of large molecules and a greater surface area compared to other starches, a higher viscosity and therefore SME compared to low-amylopectin blends like HAMS30/RPC70 is expected. This observation can be linked to Section 3.5, where WPS blends exhibited the highest gelatinization enthalpy, an indication for the largest amount of transformed amylopectin molecules.

### 3.3. Impact of Thermomechanical Treatment on Protein Solubility of RPC

#### 3.3.1. Extruded RPC

Temperature and shear during thermomechanical treatment can lead to the denaturation of proteins and to the formation of protein-linkages, which affect the extractability and solubility of proteins. With elevated temperatures, native proteins unfold and expose new reactive binding sites, that aggregate and form bonds with other proteins, polysaccharides, lipids, fiber or secondary plant metabolites [43].

The extractable protein content based on the dry matter of the extract can be used as an indirect measure for the content of other solubilized macronutrients besides the proteins present in the extracts. If, for example, starch solubilizes in NaCl at the given conditions, it will contribute to the dry matter of the extract and lead to a lower amount of extractable protein content. Moreover, the protein solubility reports the amount of solubilized protein relative to the absolute protein content in the sample before extraction.

**Figure 1:**
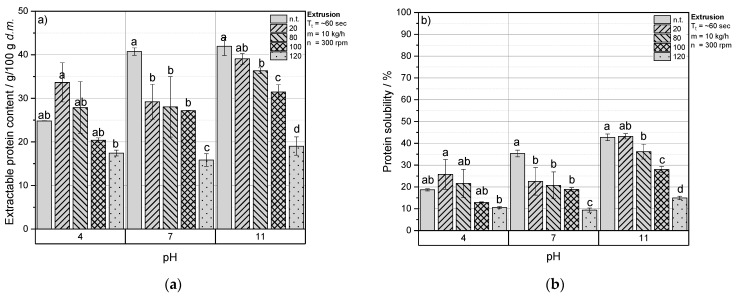
(**a**) Extractable protein content (g/100 g d.m.) and (**b**) protein solubility (%) at a pH of 4, 7 or 11 as a function of barrel temperature T_B_ of extruded rapeseed press cake (RPC). Mean values with different superscript letters comparing the effect of barrel temperature T_B_ within pH 4, 7 or 11 indicate significant differences (*p* < 0.05) based on a two-way analysis of variance (ANOVA). Where appropriate, the mean values were compared using Tukey’s honest significance test. n.t. = not treated.

Figure 1a,b shows the content of extractable protein and the protein solubility of extruded RPC at pH 4, 7 and 11 as a function of T_B_. Overall, the amount of extractable protein and the protein solubility from untreated RPC were low at an acidic pH, and were highest at strong alkaline conditions. The highest amount of extractable protein and solubility among all samples was found for untreated RPC at pH 11.

At a pH of 4, a T_B_ of 20 and 80 °C increased the content of extractable protein and solubility in RPC compared to the untreated RPC.

When T_B_ was set from 80 to 100 or 120 °C, the amount of extractable protein and the protein solubility decreased with increasing T_B_. At a pH of 7 and 11, an increasing T_B_ resulted in a decreased amount of extractable protein content and solubility. At pH 7, the extractable protein content and solubility for RPC extruded at 20 °C was significantly lower as for untreated RPC, and a significant decrease was observed when T_B_ increased from 100 to 120 °C. At pH 11, with an increase of T_B_ to 20, 80 and 100 °C, the amount of extractable protein content decreased to a similar level at each step of temperature increase; however, a significant decrease in extractable protein content was monitored when T_B_ increased from 100 to 120 °C.

Regardless of T_B_, the detectable and solubilized protein content in RPC extracts was highest for samples analyzed at pH 11.

The lower extractable protein content and solubility of untreated RPC at pH 4 compared to pH 7 and 11 is in agreement with previous research [22,47,48]. Fetzer et al. [22] observed that for cold-pressed rapeseed press cake, minimum protein solubility was observed in the acidic pH range, while the protein solubility was higher in the alkaline pH range. This can be attributed to the structural properties of the two major storage proteins in rapeseed.

The two most dominant proteins found in Brassica napus (rapeseed) are the 11S globulin cruciferin (300 kDa [49]) and the 1.7–2S albumin napin (12.5–14.5 kDa [49]), of which cruciferin constitutes 50–60% and napin 20–40% of the total protein accumulated in rapeseed. The tertiary structure of cruciferin is very pH-unstable (PI 7.2), even at ambient temperature, and unfolds at a low pH, whereas napin is stable in a wider pH range (PI 11), because of its helical secondary structure [50]. At acidic conditions (pH 4), only napins (10–16 kDa) were reported in RPC protein extracts in previous studies with minor fractions of cruciferins (≥18 kDa) [22].

Since cruciferin constitutes the major protein fraction in rapeseed, consequently, the amount of extractable protein at pH 4 is lower compared to pH 7 and 11, where cruciferin predominantly occurs in protein extracts. At high-alkaline conditions, protein hydrolysis (proteolysis) can appear, where cruciferin bands can be degraded, while napin bands are only slightly affected, which in turn increases the overall protein solubility in this pH range. The ratio of soluble cruciferin and napin can vary at neutral pH (2.1:1 to 2.6:1), wherefore pH 7 gives the most mediocre picture for the amount of extractable protein. [22].

A reduced solubilisation of proteins due to extrusion processing was described in a number of studies and was attributed to protein cross-linking induced by thermal denaturation and Maillard reactions [43,51]. Napin was shown to be more stable against denaturation in a wider temperature range than cruciferin due to some structural features [49], while cruciferin denatures at lower temperatures, indicating that the contribution of unfolding, aggregation and cross-linking of the major rapeseed storage protein cruciferin on the overall reduced protein solubility is high. Cruciferin and napin of rapeseed protein isolate showed a denaturation temperature of 84 and 102 °C in previous research [50]. It can therefore be assumed that at a T_B_ of 100 °C, cruciferin is already denatured, whereas napin might still be native or partially native. When T_B_ increased from 100 to 120 °C, it is likely that both main proteins were denatured and might have formed new protein bonds, which explains the significant decrease of extractable protein content and solubility at pH 7 and 11. The significant decrease in extractable protein at pH 4 with an increase of T_B_ from 80 to 100 °C supports these assumptions, since napin, which denatures at temperatures > 80 °C, contributes majorly to the protein fractions extractable at acidic pH.

Additionally to protein linkages that are formed due to protein unfolding and cross-linking, Maillard reactions products (e.g., (methy-) glyoxal) can induce protein cross-linking through reactions with lysyl, agrinyl or tryptophanyl residues. We observed that the RPC samples changed from a light yellow colour to brown due to thermomechanical treatment, which strengthens this hypothesis.

The amount of extractable RPC proteins at pH 7 and 11 (Figure 1a) was slightly higher as the protein content was detected in the dry RPC powder (see Table 2). This indicates that during extraction, RPC proteins dissolved well in NaCl, and NaCl-insoluble components of RPC (e.g., insoluble dietary fiber) accumulated as sediments during extract centrifugation, wherefore the dry matter composition of the extract shifted to a more protein-rich composition.

Overall, the protein solubility of RPC was lower compared to rapeseed meal investigated in previous studies [52,53]. Fetzer et al. [22] reported a protein solubility of 35, 55 and 65% for cold-pressed rapeseed meal at pH 4, 7 and 11. This might be due to the higher fat content of the RPC used in our study (23.4 g/100 g) compared to the study of Fetzer et al. (2.8 g/100 g), thus enhancing complex formation of lipids what can hinder the solubilisation of proteins [54]. Another reason can be the presence of phytic acid in the RPC used in our study. Phytic acid accumulates in large quantities in RPC and can form complexes with proteins which decrease the protein solubility [55,56].

#### 3.3.2. Thermomechanical Treatment of Rpc under Defined Conditions

**Figure 2:**
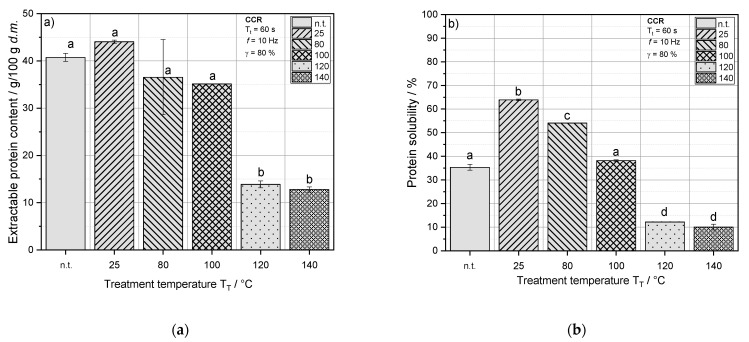
(**a**) Extractable protein content (g/100 g d.m.) and (**b**) protein solubility (%) at a pH of 7 as a function of the treatment temperature T_T_ of rapeseed press cake (RPC). Mean values with different superscript letters indicate significant differences (*p* < 0.05) based on a one-way analysis of variance (ANOVA). Where appropriate, the mean values were compared using Tukey’s honest significance test. n.t. = not treated.

Figure 2a,b illustrate the amount of extractable protein and protein solubility (pH 7) of RPC treated at defined thermomechanical conditions.

RPC exhibited a slightly higher amount of extractable protein and solubility when treated at 25 °C compared to the untreated sample. This effect was also seen for extruded RPC at a T_B_ of 20 °C and extracted at pH 4. At a T_T_ and T_B_ of 25 and 20 °C, respectively, the effect of the mechanical energy input on protein extractability of RPC proteins dominates over the impact of temperature (see extruder response reported in Table 3 for the sample RPC100). An increase of extractable protein and solubility due to mechanical input can be explained by the disruptive impact of shear on the cellular structure of RPC, wherefore the solvent during protein extraction can access a greater surface area of rapeseed components resulting in an increased content of dissolved protein in the extract.

With T_T_ > 25 °C, the proteins were less extractable and protein solubility decreased, which can be explained by the onset of protein aggregation reactions at these temperatures and a higher mobility of macromolecules due to elevated temperatures, increasing the chance for the formation of new linkages [57]. The temperature during the pressing of rapeseed oil, where RPC is generated, is continuously below 60 °C. Considering that the denaturation temperatures of the main rapeseed protein fractions napin and cruciferin are higher than 60 °C, a relatively high protein nativity of the rapeseed proteins in RPC can be assumed. Martin et al. [15] reported reaction onset temperatures of rapeseed components to be >70 °C, indicated by an increase of the complex modulus (G*) during a temperature sweep measurement in a closed-cavity rheometer. The experimental set-up and material conditions in this study were set alike the conditions in the present study, wherefore it can be assumed that with an increase of T_T_ from 25 to 80 °C, aggregation reactions of rapeseed proteins are induced, and consequently, the extractability of proteins is reduced.

At a T_T_ of 80 and 100 °C, similar amounts of protein could be extracted; however, protein solubility decreased significantly. Furthermore, significantly less protein was extracted and solubilized in RPC treated at 120 and 140 °C. This can again be linked to the high denaturation temperature of napin that is only exceeded at a T_T_ of 120 °C. Extruded samples exhibited a very similar rapid decrease of extractable protein content and solubility at a temperature of 120 °C (see Figure 1a,b).

An increase of T_T_ from 120 to 140 °C only slightly decreased the amount of extractable proteins and the protein solubility in RPC. It can be assumed that with the denaturation of napin, the major part of possible formations of new protein linkages is achieved. The slight decrease of extractable protein content at T_T_ of 140 °C may be attributed to reactions of other minor proteins in RPC, that are oleosins, lipid transfer proteins and protease inhibitors [58].

#### 3.3.3. Comparison of Extrusion-Processing and Defined Thermomechanical Conditions

Compared to extruded RPC, samples treated at defined thermomechanical conditions exhibited significantly higher protein solubility up to a treatment temperature of 100 °C. The most significant decrease of protein solubility was observed when T_B_ or T_T_ were increased from 100 to 120 °C.

At 120 °C, the protein solubility of RPC was slightly lower in extruded samples compared to samples treated at defined thermomechanical conditions. A lower protein solubility of extruded RPC compared to RPC treated under defined conditions can be attributed to the higher shear forces in the extruder barrel, leading to higher local temperatures, enhancing protein aggregation reactions. It can be assumed that at temperatures above 100 °C, thermal energy input is dominating over the impact of shear stress and causing a severe decrease of protein solubility, again correlating with the denaturation temperatures of rapeseed proteins.

### 3.4. Impact of Thermomechanical Treatment and Starch Addition on Protein Solubility of RPC

#### 3.4.1. Extractable Protein Content and Solubility of RPC/Starch Blends at Neutral pH

**Figure 3:**
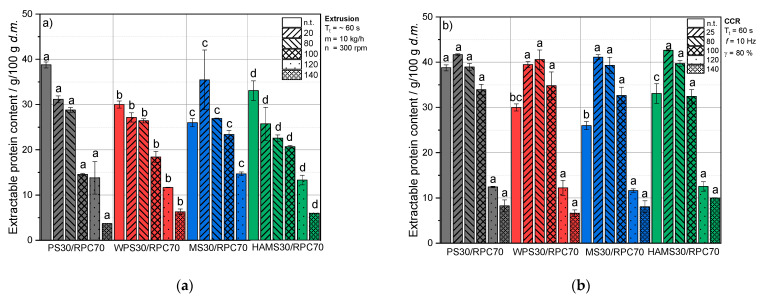

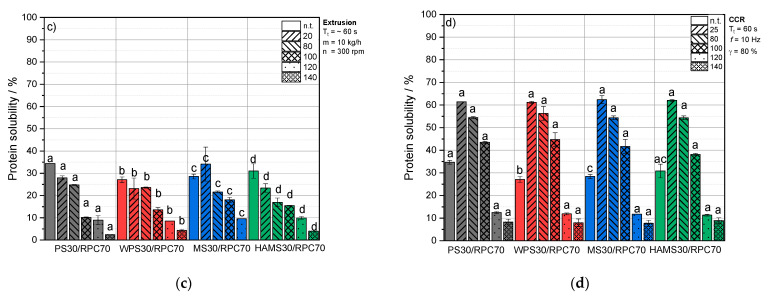
Extractable protein content (g/100 g d.m.) and protein solubility (%) at pH 7 as a function of (**a**,**c**) barrel temperature T_B_ (20, 80, 100, 120 or 140 °C) and (**b**,**d**) treatment temperature T_T_ (25, 80, 100, 120, 140) of starch/RPC blends containing 70 g/100 g w.b. RPC and 30 g/100 g w.b. potato starch (PS), waxy potato starch (WPS), maize starch (MS) or high-amylose maize starch (HAMS). Mean values with different superscript letters indicate significant differences (*p* < 0.05) between the starch types based on a two-way analysis of variance (ANOVA). Where appropriate, the mean values were compared using Tukey’s honest significance test. n.t. = not treated.

Figure 3 shows the extractable protein content and solubility of RPC/starch blends as a function of treatment temperature at a defined shear rate of 50 s^−1^.

##### Untreated Blends

The amount of extractable proteins from untreated RPC/starch blends was significantly influenced by the starch type (Figure 3a,b), and so was the protein solubility (Figure 3c,d). From untreated RPC/PS blends, the highest amount of proteins could be extracted and decreased in the order RPC/HAMS, RPC/WPS and RPC/MS. The protein solubility of untreated RPC/PS blends was equivalent to untreated RPC at pH 7 (Figure 2b), whereas blends with WPS, MS and HAMS exhibited lower protein solubility compared to RPC (Figure 3c,d).

This indicates that PS constitutes the least soluble starch among the tested varieties and accumulates as a sediment during the centrifugation step of the extraction. Accordingly, MS may exhibit a high solubility in NaCl at the given pH [59], wherefore it contributes to a larger extent to the dry matter of the extract. This in turn decreases the protein content in the extract relative to other components in the dry matter. The results of our recent studies ([14,15]), where we reported a water solubility index (%) for PS, WPS, HAMS and MS of 0.50 ± 0.00, 0.75 ± 0.35, 1.0 ± 0.14 and 1.5 ± 0.21, support these assumptions.

##### Extruded Blends

In extruded blends, the starch type had a significant impact on the extractability and solubility of proteins in all blends and at all given T_B_ (Figure 3a,c).

At 20 °C T_B_, RPC/PS and RPC/MS blends exhibited a higher protein solubility compared to RPC, whereas RPC/WPS and RPC/HAMS were equivalent to RPC. At a T_B_ of 80 °C, the protein solubility in the blends decreased in the order PS, WPS, MS and HAMS, with MS/RPC being equivalent to RPC as a pure component. At 100 °C T_B_, the protein solubility in all potato starch blends significantly decreased compared to lower temperatures, whereas RPC and maize starch blends did not show a significant decrease in protein solubility due to this temperature increase. A T_B_ of 120 °C led to equivalent protein solubilities of the RPC/starch blends compared to RPC, irrespective of starch type.

Extrusion processing at T_B_ of 20 °C decreased the extractable protein content and solubility of PS/RPC, WPS/RPC and HAMS/RPC and a T_B_ increase from 20 to 80 °C, with only slightly decreased protein extractability. In MS/RPC blends, the amount of extractable and soluble protein increased when the blends were extruded at 20 °C, but decreased as a function of increasing T_B_.

In the potato starch blends PS/RPC and WPS/RPC, a T_B_ increase from 80 to 100 °C significantly reduced the extractable protein content. This effect was greatest for PS/RPC, followed by WPS, but was only seen to a smaller extent in the maize starch blends MS/RPC and HAMS/RPC.

PS and WPS exhibit a significantly lower water-binding capacity compared to MS and HAMS, as reported in our previous studies [14,15]. This can indicate that due to severe competition on water between starches and proteins for physicochemical processes, MS and HAMS require major amounts of available process water, and can therefore limit access of water for the rapeseed proteins. Therefore, the addition of PS and WPS facilitates protein denaturation of rapeseed proteins, as indicated by a significant decrease in the protein solubility compared to MS and HAMS blends.

In extruded RPC/MS, RPC/WPS and RPC/HAMS blends, the amount of extractable protein and solubility decreased with an increase of T_B_ from 100 to 120 or to 140 °C. Compared to that, a T_B_ increase from 100 to 120 °C reduced the extractability of proteins in RPC/PS blends to a smaller extent.

##### Thermomechanical Treatment under Defined Conditions

Whereas the addition of starch to RPC had an effect on the protein solubility of extruded blends, almost no effect was detected when samples were treated under defined thermomechanical conditions.

However, at 100 °C, the protein solubility of blends containing PS, WPS and MS led to a slightly higher protein solubility compared to RPC and RPC/HAMS, and at 140 °C T_T_ the blends exhibited a slightly lower protein solubility than RPC as a pure component.

In contrast to untreated RPC/starch blends, the starch type had no significant impact on the extractability of protein in thermomechanically treated blends at all given T_T_ (Figure 3b,d).

With defined thermomechanical treatment at 25 °C, the amount of extractable protein increased significantly compared to untreated blends, irrespective of starch type (Figure 3b,d). A T_T_ increase from 25 to 80 °C slightly decreased the amount of extractable protein and solubility in all blends, and in blends with WPS a small increase of extractable protein was observed. Increasing T_T_ from 80 to 100 °C resulted in a decrease of extractable protein content and solubility in all blends.

When T_T_ increased from 100 to 120 °C, all blends exhibited significantly lower amounts of extractable protein compared to untreated blends. A further increase of T_T_ from 120 °C to 140 °C resulted again in a decrease of extractable protein and solubility. However, the extent was not as large as when T_T_ increased from 100 to 120 °C. The same effect was observed for thermomechanically treated RPC at pH 7 (Figure 2b), and can again be linked to the denaturation temperatures of cruciferin and napin [50].

##### Comparison of Extrusion-Processing and Defined Thermomechanical Conditions

Up to a T_T_ or T_B_ of 100 °C, blends treated under defined conditions exhibited higher amounts of extractable proteins and a higher solubility compared to extruded blends, as seen for the protein solubility of RPC (Figure 1b and Figure 2b). The same effect was found at a T_T_ and T_B_ of 140 °C. However, at 120 °C T_T_, extruded blends (Figure 3a,c) and blends that were treated under defined thermomechanical conditions (Figure 3b,d) exhibited similar amounts of solubilized protein.

In general, the shear rates that are applied onto the melt in the flow field of the extruder exhibit an inhomogeneous distribution due to the complex geometries of intermeshing twin-screw extruders [42]. However, the shear rate estimates made for this extruder type (see Table 1) were significantly higher than the shear rates applied in the closed-cavity rheometer. Therefore, it can be assumed that due to the high local share rates, high local temperatures are generated during extrusion-processing, inducing denaturation, rearrangement, hydrolysation or cross-linking. It can be assumed that the residence time of the blends at elevated temperatures were higher in the closed-cavity rheometer compared to the extruder, where not all barrel segments were heated to the same temperatures. However, the effect of high shear stress, and high temperatures at even a short residence time in the extruder barrel may have a larger impact on the denaturation, aggregation or formation of new protein linkages in RPC as does defined thermomechanical treatment at a constant high temperature, but with lower shear rates.

Moreover, the non-significant impact of starch type on the protein extraction and solubility in the RPC/starch blends treated under defined thermomechanical conditions can be taken as an indication that no protein–starch interactions are formed due to shear and heat under the given conditions. It is more likely that the effect of starch type on the protein extraction and solubility of extruded RPC/starch blends is due to the indistinct process conditions during extrusion, such as shear rate, diffuse mixing, viscosity, and as a consequence of that, the effect of heat transfer, which affects the denaturation, aggregation or formation of protein linkages.

#### 3.4.2. Extractable Protein Content of RPC/Starch Blends at Acidic and Alkaline pH

**Figure 4:**
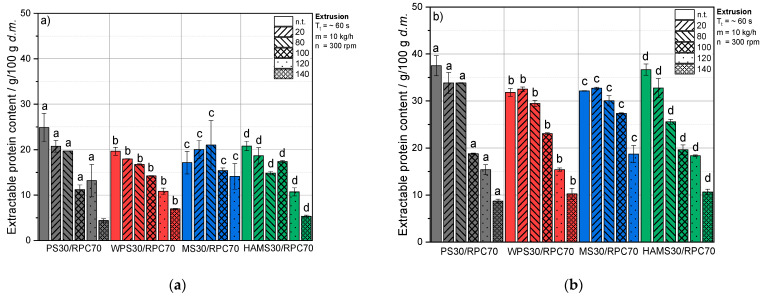
Extractable protein content (g/100 g d.m.) and protein solubility (%) at (**a**) pH 4, (**b**) pH 11 as a function of barrel temperature T_B_ (20, 80, 100, 120 or 140 °C) of starch/RPC blends containing 70 g/100 g w.b. RPC and 30 g/100 g w.b. potato starch (PS), waxy potato starch (WPS), maize starch (MS) or high-amylose maize starch (HAMS). Mean values with different superscript letters indicate significant differences (*p* < 0.05) between the starch types based on a two-way analysis of variance (ANOVA). Where appropriate, the mean values were compared using Tukey’s honest significance test. n.t. = not treated.

Figure 4 illustrates the content of extractable protein in extruded RPC/starch blends as a function of starch type, T_B_ and pH.

At pH 4, T_B_ and the starch type had a significant impact on the protein content that was solubilized after extrusion (*p* < 0.05); however, there was no significant interaction between starch type and T_B_ (*p* < 0.05), and the amount of soluble protein was lower as at pH 7 and 11 (Figure 3b and Figure 4b).

For PS, the amount of soluble protein differed only slightly between samples extruded at a T_B_ of 20 or 80 °C, and a large decrease of protein solubility was observed when T_B_ increased from 80 to 100 °C. This applied to both pH values. For MS at pH 11, the protein solubility for samples extruded at 80 and 100 °C was only slightly lower than for samples extruded at 20 °C. These two observations can be related to the low T_gel_ of PS and the comparatively high T_gel_ of MS (discussed in Section 4).

### 3.5. Impact of Extrusion on Starch Gelatinization and Hydrolysis

Table 4 reports the impact of barrel temperature and starch type on the gelatinization temperature, degree and enthalpy of extruded RPC/starch blends. T_B_ had a significant impact on T_gel_ (*p* < 0.05) and ∆H_gel_ (*p* < 0.01) of all starches, the starch types differed significantly in ∆H_gel_ (*p* < 0.01), but did not differ significantly in T_gel_ (*p* > 0.05). After extrusion, the thermal properties (∆H_gel_) of the blends were significantly lower as in the unextruded samples.

WPS blends exhibited the lowest T_gel_ and the highest ∆ H_gel_ compared to PS and MS blends. D_gel_ increased with increasing T_B_ and a D_gel_ of > 90% was found for WPS blends at a T_B_ of 80 °C, for PS blends at 100 °C and for MS blends at 120 °C. PS30/RPC70 had a higher T_gel_ than WPS30/RPC70, but though lower than MS30/RPC70. This explains that for PS and MS blends, the stage of full gelatinization, indicated by the absence of a peak in the thermogram, was completed at 100 °C and 120 °C, respectively. The ∆ H_gel_ of PS, WPS and MS blends had already decreased by 58, 50 and 81% when the blends were extruded at 20 °C compared to unextruded blends. For HAMS, no thermal peak was observed at any T_B_.

Starch gelatinization indicates the disorganization of the semi-crystalline structure into an amorphous state, and since HAMS exhibits >67% amorphous amylose and the blends only contained 30 g/100 g (w.b.) starch, the transformation of the remaining crystalline amylopectin into the amorphous state in the blends might not be detectable with the DSC. In a number of previous studies, there was also no distinct endothermic peak found for the gelatinization of high-amylose starches. Russel et al. [60] detected one broad endotherm between 66 and 104 °C during the heating of amylomaize starch containing 70% amylose conditioned to 57% water. Similar observations were made by Eberstein et al. [61]. The authors considered the DSC to be not sufficiently sensitive for the detection of gelatinization.

The high ∆H_gel_ of WPS30/RPC70, and the low T_gel_ compared to other starch types is also in accordance with the literature and can be attributed to a high water uptake and a high degree of transformation from crystalline to amorphous structures of amylopectin rich starches [30,62]. In contrast, high-amylose starches are known to exhibit a low water absorption and solubility, and an overall high resistance to gelatinization and hydrolysis [63,64]. This is in alignment with our study, where no thermal transition peak was present in HAMS30/RPC70 [65,66].

An increase in D_gel_ with an increasing extrusion temperature was reported several times in the literature for sweet potato starch [67] and maize starch [68] as single components, as well as for multicomponent biopolymers, such as bran-enriched wheat flour [69].

In the RPC/starch blends, severe competition for water likely takes place with both components requiring water for physicochemical transformations, especially since the starch and protein content in the blends was set to be relatively equal (27 and 30 g/100 g respectively). Furthermore, the fibers present in the RPC have a high capacity to hydrate, wherefore they restrict the availability of the plasticizer, increase the melt viscosity, and reduce the availability of water required for gelatinization [69]. Although there was an excess of RPC in the blends and the process water was limited to 29 g/100 g d.m., full starch gelatinization for PS, WPS and MS was observed at max. 120 °C.

**Figure 5:**
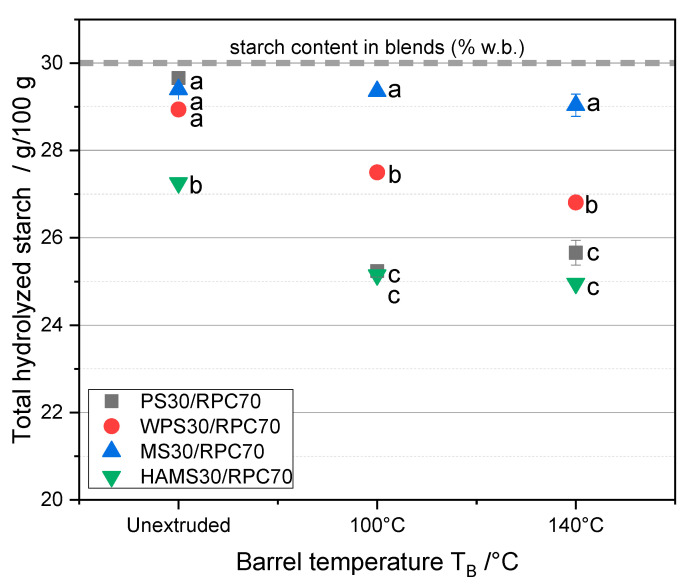
Content of total hydrolyzed starch (S_H_, g/100 g) of 70 g/100 g rapeseed press cake (RPC) mixed with 30 g/100 g potato starch (PS), waxy potato starch (WPS), maize starch (MS) and high-amylose maize starch (HAMS). Mean values with different superscript letters indicate significant differences (*p* < 0.05) between the starch types based on a two-way analysis of variance (ANOVA). Where appropriate, the mean values were compared using Tukey’s honest significance test.

As illustrated in Figure 5, the starch type and T_B_ had a significant impact on S_H_; furthermore, a significant interaction of starch type and T_B_ was observed (*p* < 0.01). In the unextruded blends, almost the whole amount of PS, WPS and MS that was present in the blends (30 g/100 g), was hydrolysed, wherefore only 27 g/100 g HAMS could be hydrolyzed. MS in extruded blends at a T_B_ of 140 °C could almost fully be hydrolyzed, whereas for PS, WPS and HAMS, S_H_ decreased with increasing T_B_. A T_B_ of 140 °C only marginally decreases S_H_ for MS, WPS and HAMS blends, and slightly increases S_H_ for the PS blend, compared to a T_B_ of 100 °C.

A decrease of S_H_ can indicate the formation of non-covalent or covalent bonds between starch and other components in the blends, such as rapeseed proteins, lipids or fibers, during extrusion [26]. Extrusion-induced starch–protein interactions have particularly been investigated in previous studies, though markedly with a focus on whey proteins [31,70,71]. Those effects can be detected by a parallel investigation of protein solubility and starch hydrolysis.

Allen et al. [31] discussed three possible reasons for reduced protein solubility when proteins are extruded in starch blends. The small protein molecules might be physically entrapped in the amylopectin matrix that is partially broken down during gelatinization. Since in our study, the reduction of protein solubility was not larger in WPS blends than in PS, MS or HAMS blends, we assume that this effect did not occur. Additionally, the authors discussed the transformation of a crystalline to a more amorphous structure, and the leakage of amylose out of the granula might provide the opportunity for proteins to align with amylose molecules in the shear zone of the extruder and stabilize due to covalent bonds after exiting the die [29,31,72]. However, the solubility of proteins extruded with HAMS (67% amylose) was not lower compared to the starches containing a higher content of amylopectin, therefore with our results, this effect could not be observed. A third theory is the formation of covalent protein–starch linkages during thermomechanical treatment, that would decrease protein solubility and the amount of hydrolyzed starch synergistically [31,72]. Since the amount of hydrolyzed starch did only slightly decrease in MS blends and to a slightly higher extent in WPS and HAMS blends, when T_B_ increased from 100 to 140 °C, but the protein solubility decreased significantly in these samples, we assume, that rather than protein–starch bonds, protein–protein bonds between unfolded rapeseed proteins are formed during extrusion that cannot be solubilized in NaCl.

The lower degree of hydrolysis of HAMS-based blends compared to MS, PS and WPS blends, regardless of whether the samples were extruded or not, may be attributed to the low susceptibility to degradation of HAMS due to its high amylose content. Some studies report that high-amylose maize starch is less susceptible to various physicochemical treatments (e.g., hydrothermal treatments) than normal or waxy starches, due to its lower crystallinity, small particle size and high surface area [66,73,74,75,76].

In our study, the extrusion-induced reduction of hydrolyzed starch content in PS30/RPC70 may indicate that the starch has interacted with rapeseed components during extrusion, making the starch less accessible for the enzymes during analysis. This observation could be corroborated by the relatively low T_gel_ and the comparatively large decrease of protein solubility at T_B_ 100 °C of PS30/RPC70, indicating that polymerization occurred from interactions between exposed binding sites from unfolded rapeseed proteins and gelatinized starch. However, since process conditions during extrusion processing were significantly influenced by the starch type, and this effect was not found in blends treated under defined thermomechanical conditions, we assume that rheological effects due to the starch type during the analysis of starch and protein solubility dominated over actual protein–starch bonds. To resolve this, future studies should systematically evaluate the impact of an isolated rapeseed protein addition to starch blends under defined thermomechanical conditions.

### 3.6. Impact of Extrusion on Total, Soluble, and Insoluble Dietary Fiber

**Figure 6:**
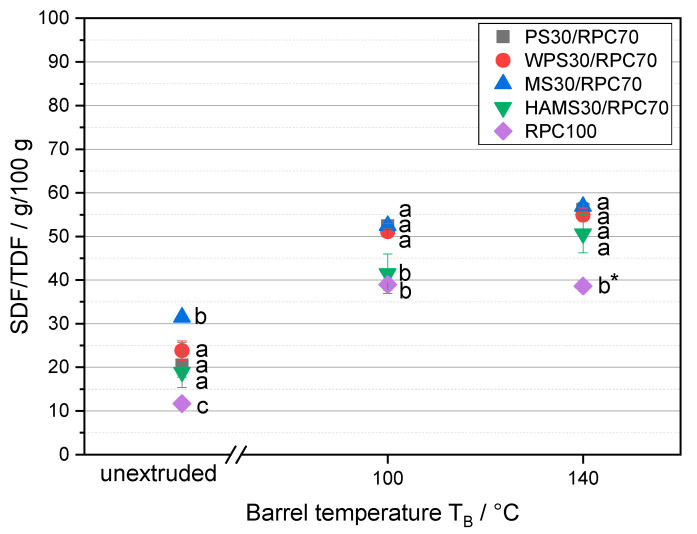
Ratio of soluble (SDF) to total (TDF) dietary fiber (SDF/TDF) of rapeseed press cake (RPC100) and RPC/starch blends containing potato starch (PS), waxy potato starch (WPS), maize starch (MS) or high-amylose maize starch (HAMS) as a function of barrel temperature T_B_ (unextruded, 100 °C, 140 °C). Mean values with different superscript letters indicate significant differences (*p* < 0.05) between the blends based on a two-way analysis of variance (ANOVA). Where appropriate, the mean values were compared using Tukey’s honest significance test. * Extruded at 120 °C.

Figure 6 shows the ratio of soluble to total dietary fiber in RPC and RPC/starch blends as a function of T_B_.

RPC exhibited the least amount of SDF relative to TDF in an unextruded and extruded condition. With the addition of starch, SDF/TDF increased in the order of RPC/HAMS, RPC/MS, RPC/WPS and RPC/PS. With an increase of T_B_ to 100 °C, the SDF/TDF of RPC increased, but an increase to 120 °C did not affect the SDF/TDF of RPC.

With a T_B_ of 100 and 140 °C, the amount of SDF increased regardless of the starch type in the blends. There was only a slight increase of SDF in the samples when T_B_ increased from 100 to 140 °C; however, SDF/TDF of RPC/HAMS increased to a larger extent.

The increase of SDF/TDF with the addition of starch to RPC can be related to the methodology of dietary fiber analysis in our study, where the first step of sample treatment is an enzymatic digestion of amylose using amylase. The presence of antinutritional components in RPC, such as phytic acid, can inhibit the starch digestion by amylase, as shown in previous research [50,77]. Furthermore, the presence of resistant starch can increase the measured SDF values, since they are not available for enzymatic hydrolysis by amylase. Blends containing HAMS consequently exhibit the least amount of soluble, and the highest amount of insoluble fractions. This effect was also seen for starch hydrolysis (Figure 5). HAMS can be considered as resistant starch, less affected by enzymatic hydrolysis than high amylopectin starches like WPS.

A number of studies based on rice bran or wheat bran described an increase of SDF/TDF due to extrusion [32,34]. An extrusion caused by a shift from insoluble to more soluble dietary fiber fractions has recently been observed by Naumann et al. [33], when lupin kernel fiber was processed in a laboratory twin-screw extruder, accompanied by a large increase of water-binding capacity. The authors associated the redistribution of IDF to SDF to mechanical rather than to thermal effects, in accordance to the studies of Ralet et al. [78].

An increase of SDF/TDF was accompanied by a high SME in the study of Naumann et al. [33]. However, in our study WPS exhibited the highest SME at a T_B_ of 100 °C and 140 °C, but the increase of SDF/TDF was not larger than in MS or HAMS, which generated lower SMEs during extrusion. The slight increase of SDF when T_B_ increased from 100 to 140 °C can be explained by the impact of thermal treatment on additional breaks of glycosidic bonds resulting in smaller and more soluble fractions of polysaccharides, as described in previous studies [79].

## 4. Conclusions

In this study, rapeseed press cake (RPC), both pure and in combination with four starch types, and varying in botanical origin and amylose content, was exposed to thermomechanical treatment at five temperatures. The impact of extrusion-processing on the solubility of rapeseed protein and fiber, hydrolyzed starch content and gelatinization properties was investigated using extraction, enzymatic and enzymatic-gravimetric analysis. Starch and protein content in the blends was equal in order to exclude an overage effect.

Indistinct process conditions (product temperatures and specific mechanical energy input) were monitored during extrusion as a function of starch type. To compensate this effect, a closed-cavity rheometer was used to expose RPC and the blends to defined thermomechanical treatment at a constant shear rate and extrusion-like temperatures. The protein solubility of the thermomechanically treated blends was analyzed accordingly.

Extrusion-processing of RPC significantly reduced the protein solubility with increasing barrel temperature, so did thermomechanical treatment at defined conditions with increasing treatment temperatures. At temperatures ≤100 °C, extruded RPC exhibited a lower protein solubility compared to thermomechanically treated RPC, whereas both treatments led to an equivalent protein solubility at 120 °C. Effects observed at temperatures ≤100 °C can be attributed to the higher shear forces and higher local temperatures in the extruder barrel promoting protein aggregation reactions. At ≥100 °C, it can be assumed that thermal energy input dominates over the influence of shear stress, wherefore extrusion and defined thermomechanical treatment resulted in equivalent protein solubilities.

The protein solubility of extruded RPC/starch blends was significantly influenced by the starch type and decreased with increased barrel temperature. No effect of starch type was detected when samples were treated under defined thermomechanical conditions. This was considered as an indication that no protein–starch interactions were formed due to shear and heat under the given conditions. It can be assumed that the effect of starch type on the protein solubility in extruded blends was predominantly due to differences in the rheological properties of the starches, leading to indistinct process conditions during extrusion, such as shear rates or local temperatures.

At temperatures of 100 and 140 °C, blends treated with defined thermomechanical conditions exhibited higher amounts of extractable proteins and a higher protein solubility compared to extruded blends, which was attributed to higher local shear rates in the extruder barrel compared to the shear rates applied in the rheometer.

Increasing barrel temperatures decreased the degree of hydrolyzed starch, and increased the degree of gelatinization, as well as the amount of soluble dietary fibers of RPC; however, the latter was not affected by the starch type.

Our findings emphasize that the closed-cavity rheometer is a suitable tool to analyze physicochemical transformations of biopolymers at defined thermomechanical conditions in order to overcome the hurdle of indistinct process conditions during extrusion, as they are significantly affected by the raw material.

## Figures and Tables

**Table 1:** Experimental set-up for extrusion-processing and treatments at defined thermomechanical conditions.

**Extrusion Processing**	**Barrel Temperature T_B_ of Barrel Segments 2–6 (°C)**					**Residence Time (s)**	**Shear Rate** $\dot{\gamma }$ **(s^−1^)**
1	20	20	20	20	20	Approx. 60	15–2430 *
2	60	80	80	80	80	Approx. 60	15–2430 *
3	60	80	100	100	100	Approx. 60	15–2430 *
4	60	80	100	120	120	Approx. 60	15–2430 *
5	60	100	120	140	140	Approx. 60	15–2430 *
**Defined Thermomechanical Treatment**	**Treatment Temperature T_T_ (°C)**					**Treatment Time T_t_ (s)**	**Shear Rate** $\dot{\gamma }$ **(s^−1^)**
1	25					60	50
2	80					60	50
3	100					60	50
4	120					60	50
5	140					60	50

* Values were investigated by Emin and Schuchmann [42] using CFD simulation based on an extruder of the same type, a similar screw configuration, and the same screw speed as used in this study. Shear rates differ largely as a function of location of the melt in the different screw sections. Therefore, values are only given as an estimation.

**Table 2:** Chemical composition of rapeseed press cake (RPC100) and blends of 30 g/100 g potato starch (PS), waxy potato starch (WPS), maize starch (MS) and high-amylose maize starch (HAMS) in combination with 70 g/100 g RPC.

Chemical Composition	RPC100	PS30/RPC70	WPS30/RPC70	MS30/RPC70	HAMS30/RPC70
Dry matter (d.m.) (%)	95.10 ± 0.03 ^a^	92.18 ± 0.01 ^b^	91.96 ± 0.08 ^b^	93.50 ± 0.06 ^c^	93.41 ± 0.03 ^c^
Protein (% d.m.)	38.20 ± 0.30 ^a^	27.40 ± 0.11 ^b^	27.38 ± 0.29 ^b^	26.99 ± 0.038 ^b^	27.28 ± 0.029 ^b^
Ash (% d.m.)	7.30 ± 0.02 ^a^	4.72 ± 0.02 ^b^	4.79 ± 0.01 ^b^	4.67 ± 0.025 ^b^	4.41 ± 0.55 ^b^
TDF (% d.m.)	35.67 ± 5.19 ^a^	10.28 ± 2.82 ^b^	9.01 ± 1.11 ^b^	11.51 ± 0.14 ^b^	16.92 ± 0.37 ^c^
SDF (% d.m.)	4.17 ± 1.55 ^a^	1.82 ± 0.13 ^b^	1.95 ± 0.05 ^b^	3.38 ± 0.11 ^c^	2.97 ± 0.28 ^c^
IDF (% d.m.)	31.51 ± 4.96 ^a^	8.47 ± 2.81 ^b^	7.06 ± 1.11 ^b^	8.14 ± 0.07 ^b^	13.95 ± 0.24 ^c^
Starch (% d.m.)	3.00 ± 0.02 ^a^	29.66 ± 0.014 ^b^	28.93 ± 0.052 ^b^	29.39 ± 0.021 ^b^	27.26 ± 0.038 ^b^
Lipid (% d.m.)	23.40 ± 0.90 ^a^	16.65 ± 0.25 ^b^	16.16 ± 0.02 ^b^	15.71 ± 0.03 ^b^	16.17 ± 0.11 ^b^
Particle size distribution (Dv_0.5_/µm)	261.1 ± 4.5 ^a^	87.0 ± 1.4 ^b^	160.7 ± 13.1 ^c^	131.5 ± 6.7 ^d^	141.1 ± 6.8 c ^e^

Mean values with different superscript letters within one row indicate significant differences (*p* < 0.05) based on a one-way analysis of variance (ANOVA). Where appropriate, the mean values were compared using Tukey’s honest significance test.

**Table 3:** Specific mechanical energy (SME) input and product temperature of extruded rapeseed press cake (RPC100) and blends of 30 g/100 g potato starch (PS), waxy potato starch (WPS), maize starch (MS) or high-amylose maize starch (HAMS) in combination with 70 g/100 g RPC as a function of barrel temperature T_B_.

		**SME (Wh/kg)**			
**T_B_ (°C) in segment 6**	RPC100	PS30/RPC70	WPS30/RPC70	MS30/RPC70	HAMS30/RPC70
20	61.23 ± 1.5 ^a^	98.13 ± 6.16 ^b^	104.32 ± 2.95 ^b^	60.68 ± 0.63 ^a^	69.16 ± 0.63 ^a^
80	38.08 ± 1.2 ^a^	76.73 ± 0.92 ^b^	83.70 ± 1.45 ^b^	47.76 ± 4.02 ^c^	53.22 ± 0.53 ^d^
100	37.37 ± 1.1 ^a^	74.89 ± 1.29 ^b^	78.52 ± 0.87 ^b^	45.85 ± 2.40 ^c^	46.25 ± 0.44 ^c^
120	36.57 ± 1.0 ^a^	72.486 ± 1.53 ^b^	74.82 ± 1.16 ^b^	45.42 ± 2.73 ^c^	40.74 ± 0.27 ^ac^
140	-	65.24 ± 2.25 ^a^	68.94 ± 1.42 ^a^	36.52 ± 1.10 ^b^	38.40 ± 0.50 ^b^
		**Product Temperature (°C)**			
**T_B_ (°C) in segment 6**	RPC100	PS30/RPC70	WPS30/RPC70	MS30/RPC70	HAMS30/RPC70
20	30.0 ± 0.5 ^a^	45.1 ± 0.5 ^b^	52.2 ± 0.5 ^ab^	43.7 ± 1.0 ^c^	42.0 ± 0.3 ^c^
80	72.1 ± 0.4 ^a^	85.5 ± 0.5 ^b^	90.2 ± 0.4 ^c^	80.8 ± 0.8 ^d^	72.0 ± 0.7 ^a^
100	88.1 ± 0.5 ^a^	104.0 ± 0.3 ^b^	100.0 ± 0.2 ^b^	98.0 ± 0.6 ^b^	90.3 ± 1.2 ^a^
120	108.2 ± 0.4 ^a^	116.1 ± 0.1 ^b^	108.8 ± 0.5 ^a^	111.3 ± 0.3 ^c^	100.0 ± 1.0 ^d^
140	-	135.3 ± 0.2 ^a^	123.4 ± 0.4 ^b^	124.3 ± 0.2 ^b^	112.3 ± 1.2 ^c^

Mean values with different superscript letters within one row indicate significant differences (*p* < 0.05) based on a one-way analysis of variance (ANOVA). Where appropriate, the mean values were compared using Tukey’s honest significance test.

**Table 4:** Degree of gelatinization D_gel_, gelatinization temperature T_gel_ and gelatinization enthalpy ∆ H_gel_ of 70 g/100 g rapeseed press cake (RPC) combined with 30 g/100 g potato starch (PS), waxy potato starch (WPS), maize starch (MS) and high-amylose maize starch (HAMS).

Barrel Temperature T_B_ (°C)	PS30/RPC70	WPS30/RPC70	MS30/RPC70	HAMS30/RPC70
**Degree of gelatinization D_gel_ (%)**				
20	58.14 ^a^	49.59 ^b^	81.71 ^c^	No peak
80	78.11 ^a^	93.31 ^b^	81.68 ^c^	No peak
100	94.97 ^a^	No peak	56.26 ^b^	No peak
120	No peak	No peak	91.39	No peak
140	No peak	No peak	No peak	No peak
**Gelatinization temperature T_gel_ (°C)**				
Not extruded	68.82 ± 0.49 ^a^	54.29 ± 0.51 ^b^	67.65 ± 0.48 ^c^	No peak
20	69.48 ± 1.08 ^a^	59.15 ± 0.49 ^b^	70.92 ± 1.24 ^c^	No peak
80	67.85 ± 0.14 ^a^	56.87 ± 0.12 ^b^	70.84 ± 1.01 ^c^	No peak
100	67.14 ± 0.11 ^a^	No peak	70.74 ± 0.88 ^b^	No peak
120	No peak	No peak	73.35 ± 0.64	No peak
140	No peak	No peak	No peak	No peak
**Gelatinization enthalpy ∆ H_gel_ (J/g)**				
Not extruded	1.74 ± 0.07 ^a^	8.12 ± 0.23 ^b^	1.16 ± 0.19 ^c^	No peak
20	0.73 ± 0.17 ^a^	4.09 ± 0.12 ^b^	0.21 ± 0.01 ^c^	No peak
80	0.38 ± 0.00 ^a^	0.54 ± 0.01 ^b^	0.21 ± 0.01 ^c^	No peak
100	0.08 ± 0.01 ^a^	No peak	0.50 ± 0.01 ^b^	No peak
120	No peak	No peak	0.09 ± 0.01	No peak
140	No peak	No peak	No peak	No peak

Mean values with different superscript letters within one row indicate significant differences (*p* < 0.05) based on a one-way analysis of variance (ANOVA). Where appropriate, the mean values were compared using Tukey’s honest significance test.

## Data Availability

No data reported.

## Abbreviations

D_gel_	Degree of gelatinization (%)
d.m.	Dry matter (g/100g)
HAMS	High-amylose maize starch
∆ H_gel_	Gelatinization enthalpy (J/g)
IDF	Insoluble dietary fiber (g/100 g)
m	Mass flow rate (kg/h)
MS	Maize starch
n	Screw speed (rpm)
n.t.	Not treated
PS	Potato starch
RPC	Rapeseed press cake
SDF	Soluble dietary fiber (g/100 g)
S_H_	Total hydrolyzed starch (g/100 g)
SME	Specific mechanical energy (Wh/kg)
T	Temperature (°C)
T_B_	Barrel temperature (°C)
TDF	Total soluble dietary fiber (g/100 g)
T_gel_	Gelatinization temperature (°C)
T_T_	Treatment temperature (°C)
T_t_	Treatment time (s)
w.b.	Wet basis (g/100 g)
WPS	Waxy potato starch

## References

[1] Hoglund E., Eliasson L., Oliveira G., Almli V.L., Sozer N., Alminger M. (2018). Effect of drying and extrusion processing on physical and nutritional characteristics of bilberry press cake extrudates. LWT Food Sci. Technol..

[2] Yagci S., Gogus F. (2008). Response surface methodology for evaluation of physical and functional properties of extruded snack foods developed from food-by-products. J. Food Eng..

[3] Da Silva A.P.L., Berrios J.D.J., Pan J., Ascheri J.L.R. (2018). Passion fruit shell flour and rice blends processed into fiber-rich expanded extrudates. CyTA J. Food.

[4] Karkle E.L., Alavi S., Dogan H. (2012). Cellular architecture and its relationship with mechanical properties in expanded extrudates containing apple pomace. Food Res. Int..

[5] Wang S.Y., Kowalski R.J., Kang Y.F., Kiszonas A.M., Zhu M.J., Ganjyal G.M. (2017). Impacts of the Particle Sizes and Levels of Inclusions of Cherry Pomace on the Physical and Structural Properties of Direct Expanded Corn Starch. Food Bioprocess. Tech..

[6] Makila L., Laaksonen O., Diaz J.M.R., Vahvaselka M., Myllymaki O., Lehtomaki I., Laakso S., Jahreis G., Jouppila K., Larmo P. (2014). Exploiting blackcurrant juice press residue in extruded snacks. LWT Food Sci. Technol..

[7] Onwulata C.I., Konstance R.P. (2006). Extruded corn meal and whey protein concentrate: Effect of particle size. J. Food Process. Pres..

[8] Onwulata C.I., Smith P.W., Konstance R.P., Holsinger V.H. (2001). Incorporation of whey products in extruded corn, potato or rice snacks. Food Res. Int..

[9] Robin F., Dubois C., Pineau N., Schuchmann H.P., Palzer S. (2011). Expansion mechanism of extruded foams supplemented with wheat bran. J. Food Eng..

[10] Nikinmaa M., Nordlund E., Poutanen K., Sozer N. (2018). From Underutilized Side-Streams to Hybrid Food Ingredients for Health. Cereal Food World.

[11] Martin A., Osen R., Greiling A., Karbstein H.P., Emin A. (2019). Effect of of rapeseed press cake and peel on the extruder response and physical pellet quality in extruded fish feed. Aquaculture.

[12] Tyapkova O., Osen R., Wagenstaller M., Baier B., Specht F., Zacherl C. (2016). Replacing fishmeal with oilseed cakes in fish feed—A study on the influence of processing parameters on the extrusion behavior and quality properties of the feed pellets. J. Food Eng..

[13] Smulikowska S., Czerwiński J., Mieczkowska A. (2006). Nutritional value of rapeseed expeller cake for broilers: Effect of dry extrusion. J. Anim. Feed Sci..

[14] Martin A., Osen R., Karbstein H.P., Emin M.A. (2021). Impact of Rapeseed Press Cake on the Rheological Properties and Expansion Dynamics of Extruded Maize Starch. Foods.

[15] Martin A., Osen R., Karbstein H.P., Emin M.A. (2021). Linking Expansion Behaviour of Extruded Potato Starch/Rapeseed Press Cake Blends to Rheological and Technofunctional Properties. Polymers.

[16] Khattab R.Y., Arntfield S.D. (2009). Functional properties of raw and processed canola meal. LWT Food Sci. Technol..

[17] Mahajan A., Dua S. (1997). Nonchemical approach for reducing antinutritional factors in rapeseed (Brassica campestris var. Toria) and characterization of enzyme phytase. J. Agric. Food Chem..

[18] Mahajan A., Dua S., Bhardwaj S. (1997). Imbibition induced changes in antinutritional constituents and functional properties of rapeseed (Brassica campestris var. toria) meal. FASEB J..

[19] Mawson R., Heaney R.K., Zdunczyk Z., Kozlowska H. (1995). Rapeseed Meal-Glucosinolates and Their Antinutritional Effects.6. Taint in End-Products. Nahrung.

[20] Lomascolo A., Uzan-Boukhris E., Sigoillot J.C., Fine F. (2012). Rapeseed and sunflower meal: A review on biotechnology status and challenges. Appl. Microbiol. Biot..

[21] DellaValle G., Colonna P., Patria A., Vergnes B. (1996). Influence of amylose content on the viscous behavior of low hydrated molten starches. J. Rheol..

[22] Fetzer A., Herfellner T., Stabler A., Menner M., Eisner P. (2018). Influence of process conditions during aqueous protein extraction upon yield from pre-pressed and cold-pressed rapeseed press cake. Ind. Crop. Prod..

[23] Leming R., Lember A. (2005). Chemical composition of expeller-extracted and cold-pressed rapeseed cake. Agraarteadus.

[24] Ancuța P., Sonia A. (2020). Oil Press-Cakes and Meals Valorization through Circular Economy Approaches: A Review. Appl. Sci..

[25] Pastor-Cavada E., Drago S.R., González R.J., Juan R., Pastor J.E., Alaiz M., Vioque J. (2011). Effects of the addition of wild legumes (Lathyrus annuus and Lathyrus clymenum) on the physical and nutritional properties of extruded products based on whole corn and brown rice. Food Chem..

[26] Day L., Swanson B.G. (2013). Functionality of Protein-Fortified Extrudates. Compr. Rev. Food Sci. Food Saf..

[27] Quevedo M., Jandt U., Kulozik U., Karbstein H.P., Emin M.A. (2019). Investigation on the influence of high protein concentrations on the thermal reaction behaviour of beta-lactoglobulin by experimental and numerical analyses. Int. Dairy J..

[28] Zhang B., Liu G., Ying D., Sanguansri L., Augustin M.A. (2017). Effect of extrusion conditions on the physico-chemical properties and in vitro protein digestibility of canola meal. Food Res. Int..

[29] Matthey F.P., Hanna M.A. (1997). Physical and Functional Properties of Twin-screw Extruded Whey Protein Concentrate–Corn Starch Blends. Lebensm Wiss Technol..

[30] Zhang W., Li S., Zhang B., Drago S.R., Zhang J. (2016). Relationships between the gelatinization of starches and the textural properties of extruded texturized soybean protein-starch systems. J. Food Eng..

[31] Allen K.E., Carpenter C.E., Walsh M.K. (2007). Influence of protein level and starch type on an extrusion-expanded whey product. Int. J. Food Sci. Technol..

[32] Andersson A.A.M., Andersson R., Jonsäll A., Andersson J., Fredriksson H. (2017). Effect of Different Extrusion Parameters on Dietary Fiber in Wheat Bran and Rye Bran. J. Food Sci. J. Food Sci..

[33] Naumann S., Schweiggert-Weisz U., Martin A., Schuster M., Eisner P. (2021). Effects of extrusion processing on the physiochemical and functional properties of lupin kernel fibre. Food Hydrocoll..

[34] Vasanthan T., Gaosong J., Yeung J., Li J. (2002). Dietary fiber profile of barley flour as affected by extrusion cooking. Food Chem..

[35] German Food Act (2005). Methods L. 16.01-2, L. 17.00-1, L. 17.00-3. BVL Bundesamt für Verbraucherschutz und Lebensmittelsicherheit.

[36] AOAC International (2016). Official Method 945.46—Ash Determination.

[37] AOAC International (1982). Official Method 962.09—Fiber (Crude) in Animal Feed and Pet. Food.

[38] Beutler H.O. (1978). Enzymatic Determination of Starch in Foods by Hexokinase Method. Starch Stärke.

[39] AACC (1999). Hydration Capacity of Pregelatinized Cereal Products. Approved Methods of Analysis.

[40] Osen R., Toelstede S., Wild F., Eisner P., Schweiggert-Weisz U. (2014). High moisture extrusion cooking of pea protein isolates: Raw material characteristics, extruder responses, and texture properties. J. Food Eng..

[41] Koch L., Emin M.A., Schuchmann H.P. (2017). Reaction behaviour of highly concentrated whey protein isolate under defined heat treatments. Int. Dairy J..

[42] Emin M.A., Schuchmann H.P. (2013). Analysis of the dispersive mixing efficiency in a twin-screw extrusion processing of starch based matrix. J. Food Eng..

[43] Liu K., Hsieh F.-H. (2008). Protein-protein interactions during high-moisture extrusion for fibrous meat analogues and comparison of protein solubility methods using different solvent systems. J. Agric. Food Chem..

[44] Ebeling M.E. (1968). The Dumas Method for Nitrogen in Feeds. J. AOAC Int..

[45] Maki K.C., Pelkman C.L., Finocchiaro E.T., Kelley K.M., Lawless A.L., Schild A.L., Rains T.M. (2012). Resistant starch from high-amylose maize increases insulin sensitivity in overweight and obese men. J. Nutr..

[46] Li M., Hasjim J., Xie F., Halley P.J., Gilbert R.G. (2014). Shear degradation of molecular, crystalline, and granular structures of starch during extrusion. Starch Stärke.

[47] Tzeng Y.-M., Diosady L.L., Rubin L.J. (1988). Preparation of Rapeseed Protein Isolates Using Ultrafiltration, Precipitation and Diafiltration. Can. Inst. Food Sci. Technol. J..

[48] Arntfield S.D., Murray E.D. (1981). The Influence of Processing Parameters on Food Protein Functionality I. Differential Scanning Calorimetry as an Indicator of Protein Denaturation. Can. Inst. Food Sci. Technol. J..

[49] Perera S.P., McIntosh T.C., Wanasundara J.P.D. (2016). Structural Properties of Cruciferin and Napin of Brassica napus (Canola) Show Distinct Responses to Changes in pH and Temperature. Plants.

[50] Tan S.H., Mailer R.J., Blanchard C.L., Agboola S.O. (2011). Canola Proteins for Human Consumption: Extraction, Profile, and Functional Properties. J. Food Sci. J. Food Sci..

[51] Chen B.Y., Yu C., Liu J.F., Yang Y.L., Shen X.C., Liu S.W., Tang X.Z. (2017). Physical properties and chemical forces of extruded corn starch fortified with soy protein isolate. Int. J. Food Sci. Tech..

[52] Salazar-Villanea S., Bruininx E.M.A.M., Gruppen H., Hendriks W.H., Carré P., Quinsac A., van der Poel A.F.B. (2016). Physical and chemical changes of rapeseed meal proteins during toasting and their effects on in vitro digestibility. J. Anim. Sci. Biotechnol..

[53] Mosenthin R., Messerschmidt U., Sauer N., Carré P., Quinsac A., Schöne F. (2016). Effect of the desolventizing/toasting process on chemical composition and protein quality of rapeseed meal. J. Anim. Sci. Biotechnol..

[54] Rommi K., Ercili-Cura D., Hakala T.K., Nordlund E., Poutanen K., Lantto R. (2015). Impact of total solid content and extraction pH on enzyme-aided recovery of protein from defatted rapeseed (Brassica rapa L.) press cake and physicochemical properties of the protein fractions. J. Agric. Food Chem..

[55] Miller N., Pretorius H.E., Du Toit L.J. (1986). Phytic acid in sunflower seeds, pressed cake and protein concentrate. Food Chem..

[56] Hídvégi M., Lásztity R. (2002). Phytic acid content of cereals and legumes and interaction with proteins. Period. Polytech. Chem. Eng..

[57] Emin M.A., Quevedo M., Wilhelm M., Karbstein H.P. (2017). Analysis of the reaction behavior of highly concentrated plant proteins in extrusion-like conditions. Innov. Food Sci. Emerg..

[58] Wanasundara J.P.D., Tan S., Alashi A.M., Pudel F., Blanchard C. (2017). Proteins from Canola/Rapeseed: Current Status.

[59] Hedayati S., Shahidi F., Koocheki A., Farahnaky A., Majzoobi M. (2016). Physical properties of pregelatinized and granular cold water swelling maize starches at different pH values. Int. J. Biol. Macromol..

[60] Russell P.L. (1987). Gelatinisation of starches of different amylose/amylopectin content. A study by differential scanning calorimetry. J. Cereal. Sci..

[61] Eberstein K., Höpcke R., Kleve, Konieczny-Janda G., Stute R. (1980). DSC-Untersuchungen an Stärke Teil I. Möglichkeiten thermoanalytischer Methoden zur Stärkecharakterisierung. Starch Stärke.

[62] Kibar E.A.A., Gönenç İ., Us F. (2010). Gelatinization of Waxy, Normal and High Amylose Corn Starches. J. Food.

[63] Ye J.P., Hu X.T., Luo S.J., Liu W., Chen J., Zeng Z.R., Liu C.M. (2018). Properties of Starch after Extrusion: A Review. Starch Stärke.

[64] Lai L.S., Kokini J.L. (1990). The effect of extrusion operating conditions on the on-line apparent viscosity of 98% Amylopectin (Amioca) and 70% Amylose (Hylon 7) corn starches during extrusion. J. Rheol..

[65] Lin L., Guo D., Zhao L., Zhang X., Wang J., Zhang F., Wei C. (2016). Comparative structure of starches from high-amylose maize inbred lines and their hybrids. Food Hydrocolloid.

[66] Tan X., Zhang B., Chen L., Li X., Li L., Xie F. (2015). Effect of planetary ball-milling on multi-scale structures and pasting properties of waxy and high-amylose cornstarches. Innov. Food Sci. Emerg..

[67] Waramboi J.G., Gidley M.J., Sopade P.A. (2014). Influence of extrusion on expansion, functional and digestibility properties of whole sweetpotato flour. Lebensm Wiss Technol..

[68] Bhatnagar S., Hanna M.A. (1994). Extrusion Processing Conditions for Amylose Lipid Complexing. Cereal Chem..

[69] Altan A., McCarthy K.L., Maskan M. (2009). Effect of Extrusion Cooking on Functional Properties and in vitro Starch Digestibility of Barley-Based Extrudates from Fruit and Vegetable By-Products. J. Food Sci. J. Food Sci..

[70] Kumar L., Brennan M.A., Mason S.L., Zheng H., Brennan C.S. (2017). Rheological, pasting and microstructural studies of dairy protein-starch interactions and their application in extrusion-based products: A review. Starch Stärke.

[71] Onwulata C.I., Tunick M.H., Thomas-Gahring A.E. (2014). Pasting and Extrusion Properties of Mixed Carbohydrate and Whey Protein Isolate Matrices. J. Food Process. Pres..

[72] Kim C.H., Maga J.A. (1987). Properties of Extruded Whey Protein Concentrate and Cereal Flour Blends. Lebensm. Wiss. Technol..

[73] Zhang B., Zhao Y., Li X., Zhang P., Li L., Xie F., Chen L. (2014). Effects of amylose and phosphate monoester on aggregation structures of heat-moisture treated potato starches. Carbohydr. Polym..

[74] Liu H., Yu L., Xie F., Chen L. (2006). Gelatinization of cornstarch with different amylose/amylopectin content. Carbohydr. Polym..

[75] Stevnebø A., Sahlström S., Svihus B. (2006). Starch structure and degree of starch hydrolysis of small and large starch granules from barley varieties with varying amylose content. Anim. Feed Sci. Tech..

[76] Leeman M.A., Karlsson M.E., Eliasson A.-C., Björck I.M.E. (2006). Resistant starch formation in temperature treated potato starches varying in amylose/amylopectin ratio. Carbohydr. Polym..

[77] Deshpande S.S., Cheryan M. (1984). Effects of Phytic Acid, Divalent Cations, and Their Interactions on Amylase Activity. J. Food Sci. J. Food Sci..

[78] Ralet M.-C., Thibault J.-F., Della Valle G. (1990). Influence of extrusion-cooking on the physico-chemical properties of wheat bran. J. Cereal Sci..

[79] Bader Ul Ain H., Saeed F., Ahmed A., Asif Khan M., Niaz B., Tufail T. (2019). Improving the physicochemical properties of partially enhanced soluble dietary fiber through innovative techniques: A coherent review. J. Food Process. Preserv..

